# Electronic medical record-based multicondition models to predict the risk of 30 day readmission or death among adult medicine patients: validation and comparison to existing models

**DOI:** 10.1186/s12911-015-0162-6

**Published:** 2015-05-20

**Authors:** Ruben Amarasingham, Ferdinand Velasco, Bin Xie, Christopher Clark, Ying Ma, Song Zhang, Deepa Bhat, Brian Lucena, Marco Huesch, Ethan A. Halm

**Affiliations:** Parkland Center for Clinical Innovation, 8435 Stemmons Freeway, Suite 1150, Dallas, TX 75247 USA; Division of General Internal Medicine, Department of Internal Medicine, University of Texas Southwestern Medical Center, Dallas, USA; Texas Health Resources, Dallas, USA; Division of Biostatistics, Department of Clinical Sciences, University of Texas Southwestern Medical Center, Dallas, USA; Office of Quality Improvement and Safety, University of Southern California Price School of Public Policy, Los Angeles, USA; Schaeffer Center for Health Policy & Economics, University of Southern California Price School of Public Policy, Los Angeles, USA; Department of Community and Family Medicine, Duke University School of Medicine, Durham, USA; Duke Fuqua School of Business, Durham, USA

**Keywords:** Readmission, Predictive model, All-cause readmission, Electronic medical record

## Abstract

**Background:**

There is increasing interest in using prediction models to identify patients at risk of readmission or death after hospital discharge, but existing models have significant limitations. Electronic medical record (EMR) based models that can be used to predict risk on multiple disease conditions among a wide range of patient demographics early in the hospitalization are needed. The objective of this study was to evaluate the degree to which EMR-based risk models for 30-day readmission or mortality accurately identify high risk patients and to compare these models with published claims-based models.

**Methods:**

Data were analyzed from all consecutive adult patients admitted to internal medicine services at 7 large hospitals belonging to 3 health systems in Dallas/Fort Worth between November 2009 and October 2010 and split randomly into derivation and validation cohorts. Performance of the model was evaluated against the Canadian LACE mortality or readmission model and the Centers for Medicare and Medicaid Services (CMS) Hospital Wide Readmission model.

**Results:**

Among the 39,604 adults hospitalized for a broad range of medical reasons, 2.8 % of patients died, 12.7 % were readmitted, and 14.7 % were readmitted or died within 30 days after discharge. The electronic multicondition models for the composite outcome of 30-day mortality or readmission had good discrimination using data available within 24 h of admission (*C* statistic 0.69; 95 % CI, 0.68-0.70), or at discharge (0.71; 95 % CI, 0.70-0.72), and were significantly better than the LACE model (0.65; 95 % CI, 0.64-0.66; *P* =0.02) with significant NRI (0.16) and IDI (0.039, 95 % CI, 0.035-0.044). The electronic multicondition model for 30-day readmission alone had good discrimination using data available within 24 h of admission (*C* statistic 0.66; 95 % CI, 0.65-0.67) or at discharge (0.68; 95 % CI, 0.67-0.69), and performed significantly better than the CMS model (0.61; 95 % CI, 0.59-0.62; *P* < 0.01) with significant NRI (0.20) and IDI (0.037, 95 % CI, 0.033-0.041).

**Conclusions:**

A new electronic multicondition model based on information derived from the EMR predicted mortality and readmission at 30 days, and was superior to previously published claims-based models.

**Electronic supplementary material:**

The online version of this article (doi:10.1186/s12911-015-0162-6) contains supplementary material, which is available to authorized users.

## Background

To encourage hospitals to improve care provided to inpatients, two key quality of care outcomes have been operationalized by the Centers for Medicare and Medicaid Services (CMS). 30-day risk-adjusted mortality and 30-day risk-adjusted readmission rates are both publicly reported and hospitals face substantial financial penalties for poor performance as part of the CMS Hospital Readmissions Reduction Program [1, 2]. Despite some evidence that a combination of careful discharge planning, provider coordination and intensive counseling can prevent re-hospitalization, success has been difficult to achieve and sustain [1–4]. Enrolling all patients into a uniform, high intensity care transition program requires a depth of case management and outpatient resources out of reach for many health systems. Accordingly, there is increasing interest in predicting patient risk of readmission [5–10], identifying high-risk patients early in the admission [7, 10], and establishing multi-disciplinary programs that target hospital and community resources in order to reduce readmissions in this high-risk subset [11].

Existing models in the literature are mainly based on administrative data and can only be used after patient discharge. In addition, they are often limited to specific disease conditions (e.g. congestive heart failure) or a subset of patients (e.g., Medicare patients). These characteristics limit the potential use of these models in practice. To be effective, programs require predictive models that adequately discriminate between high and low risk patients with a wide range of disease conditions and demographic profiles early in the hospitalization. Predictive models should enable simultaneous comparison across patients in a hospital, reduce time-consuming manual chart review by front-line staff, and work across diverse patient and hospital populations. More recent models, such as the HOSPITAL [9] model and the PREADM [10] model, included the entire adult populations or used EHR data (the PREADM model), and showed promising discriminatory power. We aim to continue to advance in this direction, but to focus on a different outcome, a composite outcome of readmission or death from any cause within 30 days of discharge.

The aim of this study was to use data from 7 diverse hospitals in one large metropolitan area that used a common commercially available electronic medical record (EMR) to: 1) construct and validate an electronic multicondition model (e-model) of all-cause 30-day readmission or mortality risk using data present in the first 24 h of admission (24-h e-model), 2) assess the incremental predictive power of the model by adding information available on hospital discharge (e.g. length of stay, other comorbidities) (discharge e-model), and 3) examine the performance of these e-models compared to two widely cited, administrative claims-based multicondition all-cause readmission models-- the LACE [6] and the CMS Hospital Wide Readmission models [12].

## Methods

The study population consisted of all consecutive patients admitted for any medical reason to any of the internal medicine services at 7 hospitals in the Dallas Fort Worth area between November 1, 2009 and October 30, 2010. Patients who left the hospital against medical advice, died during the inpatient stay, or were transferred to another acute care facility were excluded. For patients with multiple index admissions, only the first admission was included (Additional file 1: Figure S1). The seven hospitals are part of three health systems: Parkland Health & Hospital System (PHHS, a public, safety net hospital), University of Texas Southwestern (UTSW, a university teaching hospital), and Texas Health Resources (THR, a faith-based, nonprofit health system).

These health systems were chosen because they use the Epic EMR (EPIC Systems Corporation, Verona, WI), but differ in financial and operating models, teaching status, patient mix, patient volume, size, bed count, and overall mission. These health systems serve a diverse patient population that is more representative of the patients in most US hospitals than those in existing models. Each health system independently extracted data from their EMRs, generated standardized variables, and de-identified data sets prior to analysis. After the datasets were de-identified, they were consolidated and patients were randomly split into derivation and validation cohorts (50-50 split). The study was reviewed and approved by the Institutional Review Boards of UTSW and THR.

### Patient-level outcomes

The outcomes of interest were a composite of readmission or death from any cause within 30 days of discharge. Readmission was defined as non-elective re-hospitalization for any cause to any of the 75 acute care hospitals in the larger North Texas region (which includes but extends beyond the Dallas-Forth Worth metropolitan area) using a data linkage service available through the Dallas Fort Worth Hospital Council. Each health system provided data on known deaths within 30 days of discharge based on their own EMR and administrative datasets. We used information on documented encounters in each health system after 30 days post discharge to rule out deaths within 30 days. In the absence of health system evidence of survival beyond 30 days, we queried the Social Security Death Index to identify additional deaths.

### Derivation of the models predicting the 30-day readmission or death and 30-day readmission

The 30-day readmission or death model (composite outcome) was constructed using candidate risk factors that met three criteria: (1) available in the EMR at each of the participating hospital, (2) routinely collected or available within the first 24 h of hospital presentation, and (3) plausible predictors of adverse outcomes based on clinical expertise and existing literature [6–17]. Candidate variables included clinical data such as vital signs, laboratory orders and results, and comorbidities; demographic variables such as age, gender, and marital status; and prior or current healthcare utilization such as number of prior emergency room visits to the hospital. We were not able to include many social and environmental factors associated with readmission risk because they were not consistently available in every institution’s EMR [7, 12–20].

Model building occurred in five stages. First, univariate relationships between the composite outcome and each of the 30 candidate variables were assessed in the derivation cohort using a pre-specified significance threshold of *P* = 0.05. Continuous laboratory and vital sign values were transformed into categorical variables with multiple discrete levels using recursive partitioning [21]. Study team clinicians examined these cut-off values post hoc to ensure consistency with clinical interpretation. Second, to protect against over-fitting (i.e., limiting the number of predictors according to the general guideline of having 10 outcomes for each independent variable used in the multivariate model), the number of predictor variables were restricted to that estimated through a heuristic shrinkage formula [22]. Third, candidate variables were ranked by *P* value using bootstrapping with replacement in 1000 multivariate logistic regression iterations [21]. Fourth, again using a pre-specified significance threshold of P = 0.05 as well as post hoc clinical judgment, the final 27 model variables were selected to fit the model. Finally, an additional ‘discharge’ model was derived using 3 updated, supplementary variables available at time of discharge (i.e. length of stay, additional coded diagnoses including comorbidities used by CMS readmission models and those used for AHRQ Patient Safety Indicators, and an end of stay Charlson comorbidity index). Missing values occurred to various extents for lab variables, ranging from less than 2 % for vital signs to around 30 % for more selective labs (Albumin). No imputation was employed for missing values. Instead, categories for missing values were created for each variable and outcome rates were compared across the levels and pooled into the reference group. The electronic 30-day readmission model used the same variables identified in the fifth stage of the above process, and was estimated separately in the derivation cohort.

### Comparison models

For comparison with published prediction models, the Canadian LACE claims-based model and the CMS Hospital Wide Readmission (HWR) claims-based model were used. The LACE model is a multicondition model for adults of all ages designed to predict 30-day mortality or unplanned readmission among patients discharged from 11 hospitals in Ontario, Canada [6]. LACE uses length of stay, acuity of admission, Charlson comorbidity score, and prior ED visits to construct a prediction index. The CMS HWR readmission measure is a multicondition model that is conditional on the primary diagnosis. It uses the same set of comorbidity variables, but has different sets of odds ratios depending on primary diagnosis. The CMS model is designed to profile hospital performance among Medicare patients hospitalized for multiple disease conditions, using claims based data for risk adjustment and model categorization [12]. Model comparison cohorts excluded patients with psychiatric conditions and cancer as these were not included in the CMS HWR model. We do note that the CMS model was developed to evaluate hospital performance, and does not include a number of variables we used in developing our model, including previous hospitalizations, ER use, and payment source. We decided to use the CMS model as a benchmark despite these differences because it is the most widely recognized multicondition readmission model in the United States.

### Statistical analyses

Model calibration was evaluated using the Hosmer-Lemeshow *Χ*^2^ goodness of fit test and using the calibration plot (Additional file 1: Figure S3) [23]. Using interval end-points determined by the derivation cohort, five risk categories (1 = very low to 5 = very high) were created based on quintiles of predicted 30 day risk and were graphically assessed by comparing derivation and validation cohort results.

Model discrimination was assessed using several complementary methods. The *C* statistic was calculated for each fitted model, and compared between models. To provide information beyond changes in the *C* statistic, we calculated the estimated Integrated Discrimination Improvement (IDI) [24].

For the electronic 30-day readmission risk model, these methods were supplemented with classification and reclassification analyses. Classification analyses compared the electronic model’s patient-level predictions of 30-day readmission with observed readmission events.

In the reclassification analysis [24] for both the electronic model and for the CMS HSR model, the patient-level predicted probabilities of the event were ranked from highest risk to lowest risk and grouped into respective quintiles. The rankings of each model were compared to understand whether each model classified the same patients in different risk strata. All reported *P* values were based on two-tailed tests with significance level of 0.05, and no corrections for multiple comparisons were made to minimize the errors of interpretation [25]. All analyses were conducted using STATA statistical software (version 10.0; STATA Corp, College Station, TX) and RTREE (from https://pypi.python.org/pypi/Rtree/).

## Results

A total of 39,604 index admissions formed the derivation and validation cohorts (Additional file 1: Figure S1). Table 1 lists key characteristics and outcomes of this adult population. The mean age was 61.3 years but ranged from 18 to 89 (age was censored at 89 for de-identification purpose). Of these, 1169 (3 %) died within 30 days, 5142 (13 %) were readmitted within 30 days, and 6022 (15 %) were either readmitted or dead within 30 days (not exclusive).

**Table 1:** Characteristics of patients in the derivation and validation cohorts

Characteristic^a^		Derivation (n = 19 831)	Validation (n = 19 773)
Age, y (mean)		61.3 (17.7)	61.1 (17.5)
Male		9 207 (46.4)	9 182 (46.4)
Race	White	12 361 (62.3)	12 344 (62.4)
	Black	3 911 (19.7)	3 985 (20.2)
	Hispanic	2 762 (13.9)	2 670 (13.5)
Payor,	Medicare	8 191 (41.3)	8 005 (40.5)
	Medicaid	1 371 (6.9)	1 414 (7.1)
	Commercial	7 556 (38.1)	7 473 (37.8)
	Self-Pay	944 (4.8)	1 019 (5.2)
Elective admission		2 823 (14.2)	2 770 (14.0)
At least 1 prior hospitalization in past year		4 495 (22.7)	4 489 (22.7)
At least 1 prior ED visit in past year		5 653 (28.5)	5 715 (28.9)
Number of emergency contacts in EMR (up to 5)			
0		643 (3.2)	654 (3.3)
1		16 107 (81.2)	16 000 (80.9)
2		2 846 (14.4)	2 865 (14.5)
3 – 5		235 (1.2)	254 (1.3)
Principal diagnosis disease category			
Cardiorespiratory condition		2 631 (13.3)	2 482 (12.6)
Cardiovascular condition		2 425 (12.2)	2 549 (12.9)
Neurologic condition		1 104 (5.6)	1 098 (5.5)
Comorbidities (available within 24 h of admission)^b^			
Coronary atherosclerosis or angina, cerebrovascular disease		2 423 (12.2)	2 518 (12.7)
Diabetes mellitus		2 254 (11.4)	2 317 (11.7)
Congestive heart failure		1 504 (7.6)	1 474 (7.5)
Iron deficiency		1 344 (6.8)	1 380 (7.0)
Acute renal failure		1 261 (6.4)	1 237 (6.3)
Disorders of fluid, electrolyte, acid-base		1 214 (6.1)	1 241 (6.3)
Arrhythmias		1 205 (6.1)	1 204 (6.1)
Psychiatric disease		979 (4.9)	990 (5.0)
Chronic obstructive pulmonary disease		766 (3.9)	782 (4.0)
Pneumonia and other infectious diseases		737 (3.7)	745 (3.8)
Laboratory results/vitals within 24 h of admission, median (IQR)			
Albumin		3.5 (3.1 – 3.9)	3.6 (3.1 – 3.9)
Creatinine		1 (0.8 – 1.4)	1 (0.8 – 1.4)
Hematocrit		37.7 (33.4 – 41.3)	37.6 (33.3 – 41.2)
Potassium		4.1 (3.8 – 4.5)	4.1 (3.8 – 4.5)
Respiratory rate		20 (20 – 24)	20 (20 – 24)
Systolic blood pressure		155 (139 – 176)	154 (139 – 176)
Reported pain level on scale of 0 – 10		0 (0 – 4)	0 (0 – 4)
Outcomes			
30-day mortality		621 (3.1)	548 (2.8)
30-day readmission		2 638 (13.3)	2 504 (12.7)
30 day readmission or mortality		3 116 (15.7)	2 906 (14.7)
Length of stay, median (IQR)		4 (2 – 7)	4 (2 – 6)

Abbreviations: EMR, electronic medical record; IQR, interquartile range. ^a^Numbers (percent) unless otherwise stated ^b^Comorbidities for admissions in the prior year, not index admission, coded using ICD-9 diagnoses

Candidate risk predictors for both the 30-day composite outcome and 30-day readmission electronic model (e-model) are shown in Additional file 1: Table S1, and the risk predictors included in the final e-model are shown in Table 2. Derivation and validation cohorts were highly concordant across the risk spectrum (Additional file 1: Figure S2) and the cohort models were well-calibrated (Additional file 1: Figure S3). Using interval end-points determined by the derivation cohort, quintiles of predicted risk were created which ranged between 5 % and 30 % and these were concordant between derivation and validation cohorts (Additional file 1: Figure S2).

**Table 2:** Final electronic multicondition multivariate model of risk of 30 day readmission or death^a^

Risk factor	Adjusted odds ratio (95 % C.I.)	*P* value
SpO2 < = 94	1.11 (1.02 - 1.21)	0.012
BUN < = 20	0.84 (0.77 - 0.92)	0.000
Systolic BP < = 100	1.12 (1.03 - 1.22)	0.007
Diastolic BP < = 62	1.37 (1.05 - 1.79)	0.019
Pulse > 99	1.29 (1.19 - 1.40)	0.000
Sodium > 145	1.48 (1.16 - 1.88)	0.001
BNP > 2400 or NT proBNP > 18000	1.28 (1.02 - 1.61)	0.034
Anion Gap > 18	1.57 (1.19 - 2.06)	0.001
Albumin < = 2	1.83 (1.34 - 2.50)	0.000
Albumin 2 – 3	1.45 (1.31 - 1.61)	0.000
CO2 > 30	1.34 (1.10 - 1.64)	0.004
CPK < = 60	1.20 (1.07 - 1.35)	0.002
HCT > 35	0.80 (0.74 - 0.87)	0.000
Lymphocyte < = 1.3	1.11 (1.01 - 1.21)	0.023
MCV > 100	1.24 (1.03 - 1.49)	0.022
Platelets < = 90	1.51 (1.24 - 1.84)	0.000
Platelets > 350	1.32 (1.16 - 1.49)	0.000
PT > 35	2.05 (1.42 - 2.96)	0.000
TSH > 7	1.48 (1.11 - 1.98)	0.008
AST > 40	1.28 (1.14 - 1.42)	0.000
Medicare payor	1.19 (1.08 - 1.31)	0.000
Medicaid payor	1.79 (1.55 - 2.08)	0.000
Male	1.14 (1.05 - 1.24)	0.001
Elective admission status	0.67 (0.59 - 0.77)	0.000
Prior ED visits in past year	1.03 (1.02 - 1.05)	0.000
Prior hospitalizations in past year	1.17 (1.13 - 1.21)	0.000
Age	1.01 (1.01 - 1.02)	0.000
Charlson Comorbidity index	1.09 (1.07 - 1.12)	0.000

Abbreviations. BUN, blood urea nitrogen; BP, blood pressure; BNP, B-natriuretic peptide; CO_2_, carbon dioxide; CPK, creatinine kinase; TSH, thyroid stimulating hormone; AST, aspartate aminotransferase; HCT, hematocrity; MCV, mean cell volume ^a^Derivation cohort; composite outcome of 30-day readmission or mortality

For the composite outcome, the 24-h e-model had a *C* statistic of 0.69 (95 % CI: 0.68 – 0.70) which improved only modestly after the updating of comorbidities in the Charlson comorbidity index and adding length of stay, which were both available on discharge (0.71; 95 % CI: 0.70-0.72; *P* = 0.05). Model fit was adequate with generalized *R*^*2*^ of 0.06.

Model comparison with the LACE model was performed in a cohort subset (N = 17,233 patients) of the validation cohort (N = 19,773) to exclude patients with cancer or psychiatric disease. For comparison against the CMS model, a cohort subset (N = 16,937 patients) that excluded those who died within 30 days of discharge was used.

In comparison to the LACE model (Table 3) which used data available at discharge, the discharge e-model had significantly better discrimination (*C* statistic: 0.71; 95 % CI: 0.70-0.72 versus 0.65, 95 % CI: 0.64 – 0.66; difference in model fit 0.06, 95 % CI: 0.05-0.07; *P* = 0.02). The e-model also created a broader spread of predicted risk across deciles (from 4.9 % to 40.2 % compared to the LACE model (6.1 % to 32.7 %).

**Table 3:** Comparison of performance of discharge 30-day composite readmission or mortality risk models (N = 17233)^a^

	*C* statistic (95 % CI)	Generalized *R* ^2^	Predicted event rate by decile of predicted risk, %		NRI index^c^	IDI index^d^
			Lowest	Highest		
E-Risk Model^e^	0.71 (0.70-0.72)	0.070	4.9	40.2		0.082
LACE Model	0.65 (0.64-0.66)	0.052	6.1	32.7		0.042
Difference (95 % CI)	0.056 (0.047-0.066)	0.018			0.156	0.039 (0.035-0.044)
*P* value	< .001	N/A^b^			< .001	< .05

Abbreviations: CI, confidence interval; NRI, net reclassification improvement; IDI, integrated discrimination improvement; E-Risk Model, automated real-time model to identify adult medicine patients at risk for 30-day readmission using electronic medical record data; CMS-HWR, Centers for Medicare and Medicaid Services – Hospital Wide Readmission with Medicine, Cardiovascular, Cardiorespiratory, and Neurology submodels

^a^Based on model comparison cohort of 16 937 patients

^b^No test of significance applicable to difference in generalized *R* ^*2*^ between these non-nested prediction models

^c^Net reclassification improvement is the sum of the proportion of patients moving up less the proportion moving down, among patients who are readmitted, and the proportion of patients moving down less the proportion moving up, among patients who are not readmitted

^d^Discrimination slope is difference of estimated mean probabilities for events and nonevents

^e^Discharge version: Updated for length of stay, additional diagnosed comorbidities and complications

The e-model for 30-day readmission used the same variables as the composite outcome model, with different estimated odds ratios (Additional file 1: Table S2). Discrimination for the discharge e-model (0.68; 95 % CI: 0.67-0.69) was not significantly better than the 24-h e-model (0.66; 95 % CI: 0.65-0.67). The discharge e-model had similarly good fit in the validation cohort (Table 4). This model’s classification performance was adequate with sensitivity of 49 % and positive predictive values of 21 % when predictions were dichotomized as predicting an outcome if predicted probability of readmission was greater than the 70^th^ percentile (Additional file 1: Table S3).

**Table 4:** Comparison of performance of discharge 30-day readmission risk models^a^

	*C* statistic (95 % CI)	Generalized *R* ^2^	Predicted event rate by decile of predicted risk, %		NRI index^c^	IDI index^d^
			Lowest	Highest		
E-Risk Model^e^	0.68 (0.67-0.69)	0.059	5.9	30.7		0.049
CMS-HWR Model	0.61 (0.59-0.62)	0.011	9.6	20.3		0.012
Difference (95 % CI)	0.075 (0.061-0.089)	0.047			0.198	0.037 (0.033-0.041)
*P* value	< .001	N/A^b^			< .001	< .05

Abbreviations: CI, confidence interval; NRI, net reclassification improvement; IDI, integrated discrimination improvement; E-Risk Model, automated real-time model to identify adult medicine patients at risk for 30-day readmission using electronic medical record data; CMS-HWR, Centers for Medicare and Medicaid Services – Hospital Wide Readmission with Medicine, Cardiovascular, Cardiorespiratory, and Neurology submodels

^a^Based on model comparison cohort of 16 937 patients

^b^No test of significance applicable to difference in generalized *R* ^*2*^ between these non-nested prediction models

^c^Net reclassification improvement is the sum of the proportion of patients moving up less the proportion moving down, among patients who are readmitted, and the proportion of patients moving down less the proportion moving up, among patients who are not readmitted

^d^Discrimination slope is difference of estimated mean probabilities for events and nonevents

^e^Discharge version: Updated for length of stay, additional diagnosed comorbidities and complications

In comparison with the CMS HWR model (Table 4), the discharge readmission e-model had better discrimination (0.68; 95 % CI: 0.67-0.69 versus 0.61, 95 % CI: 0.59-0.62; difference 0.08, 95 % CI: 0.06-0.09; *P* < 0.01). Other measures of model discrimination improvement were confirmed by the statistical superiority of the e-model with a significant NRI (0.20) and significant improvement in the IDI (0.037; *P* < 0.05). The e-model also created a broader spread of predicted risk across the deciles (from 5.9 to 30.7 % compared to the CMS model (9.6 to 20.3 %, Table 4). Of the 2155 patients with readmissions, the e-model also correctly reclassified a greater number of patients with readmissions (873, or 40.5 %) into a higher quintile compared to the CMS model.

The reclassification analysis in Table 5 confirmed this. Using the e-model would result in significantly more accurate risk stratification than the CMS model, as the readmission rates for patients in areas of disagreement were more consistent with rates predicted by the e-model than the CMS model. For example, the readmission rate for the 231 patients predicted to be in the top 20 % by the e-model and bottom 20 % in the CMS model was high (22.5 %), whereas the readmission rate for the 81 patients predicted to be in the bottom 20 % by the e-model and top 20 % by the CMS model was very low (3.7 %). The e-model therefore was more accurate and better calibrated than the CMS model, especially when the two models differ substantially.

**Table 5:** Risk stratification comparison between discharge 30-day readmission risk e-model and CMS-HWR models

Patient risk ranking and readmission rates based on CMS-HWR model						
Based on e-Model	Top 20 %	60-80 percentile	40-60 percentile	20-40 percentile	Bottom 20 %	Total
Top 20 %	1620 (26.4 %^a^)	732 (27.2 %)	459 (19.0 %)	346 (21.1 %)	231 (22.5 %)	3388 (24.7 %)
60-80 percentile	911 (17.3 %)	836 (14.1 %)	598 (14.4 %)	543 (16.2 %)	499 (14.2 %)	3387 (15.4 %)
40-60 percentile	515 (12.8 %)	726 (12.0 %)	739 (12.4 %)	687 (12.1 %)	721 (9.2 %)	3388 (11.6 %)
20-40 percentile	261 (12.3 %)	661 (6.8 %)	751 (7.1 %)	800 (5.9 %)	914 (7.4 %)	3387 (7.2 %)
Bottom 20 %	81 (3.7 %)	432 (8.3 %)	841 (4.8 %)	1011 (4.0 %)	1022 (3.7 %)	3387 (4.6 %)
Total	3388 (20.2 %)	3387 (14.3 %)	3388 (10.6 %)	3387 (9.8 %)	3387 (8.7 %)	16,937 (12.7 %)

^a^Readmission rate. This cell means that of the 16,937 patients, 1620 were stratified into the top 20 % by both models, and the readmission rate of these 1620 patients was 26.4 %

## Discussion

In a study population comprising 7 diverse hospitals and 39,604 adults of all ages hospitalized for a broad range of medical reasons, an electronic model utilizing EMR data routinely available within 24 h of admission identified patients at high risk of post-discharge death or readmission events early in their hospitalization.

Adding information available on discharge (e.g. length of stay and other comorbidities) to the electronic model had a small incremental benefit in predicting the risk of readmission and death, but no significant impact on predicting the risk of readmission alone. This suggests that meaningful patient-level risk stratification of readmission risk can occur early in the hospital stay without waiting for further information at time of discharge. The electronic model does not require manual computation by staff and was constructed such that it can be calculated directly from the commonly used commercial EMR employed by this diverse group of 7 hospitals. With wide-spread adaption of EMR systems in US hospitals, accurate, real-time, automated prediction models have the potential to significantly improve patient care during and after hospitalization.

The present study suggests that multicondition electronic models also perform well and may be a more efficient and generalizable approach to predicting risk of readmission across a broad range of medical reasons for hospitalization. Much of the work to date has focused on disease-specific models for conditions such as heart failure, which though the most common reason for admission, still only comprise a few percent of all hospitalizations. A multicondition model would also be more practically useful compared to a spectrum of disease specific models, as many patients have multiple comorbidities.

In contrast to the CMS HWR measure, which is both claims-based and specific to elderly Fee-For-Service Medicare beneficiaries, our new electronic model should be more generalizable because it was derived and validated in a population of all ages (ranging from 18 to 89) and with a diverse payer mix including patients with commercial, Medicare, and Medicaid insurance, as well as those who are uninsured. Among those individual with Medicare, we included those with both Medicare Fee-For-Service and Medicare managed care. Similarly, this electronic model was validated in the high readmission rate environment of US healthcare. In contrast, the LACE model was validated in a lower readmission rate setting in Canada and performed poorly when used in a United Kingdom population differing from the Canadian population [26].

These findings suggest several implications for care delivery and clinical practice. The most practical and promising advantage of this new, multicondition electronic model is that it is based on data readily available in commercial EMRs in the first day of admission, so there is an opportunity to identify high risk patients in real-time early in the hospitalization. We have previously shown that it is feasible to implement a real-time, electronic heart failure model to identify high risk patients, and then target hospital and outpatient evidence-based interventions using existing hospital resources [11]. In this controlled before-and-after trial, implementation of this e-model resulted in unadjusted readmission rates declining from 26.2 to 21.2 % (*P* < 0.01) over two years, corresponding to a significant adjusted odds ratio of 0.73 (95 % CI: 0.58-0.93). These significant reductions in the readmission rate for the overall heart failure population were accomplished by intervening in about one-fifth of heart failure cases.

Readmission to a hospital within 30 days can be a marker of poor quality of care, but efforts to reduce such events often involve intensive resource management applied to all patients, or interventions that are timed too late in the admission to support effective multi-disciplinary efforts [27]. Methods that can identify those at the highest risk of adverse events and allow sufficient time to initiate and coordinate the concentration of scare resources on those most likely to benefit have great potential for accomplishing the ‘triple aim’ of higher quality, more cost-conscious care for patients and populations.

This study has several strengths and limitations worth noting. First, patients in the study population came from three very different health systems in the fourth largest metroplex in the US, which serve a large, diverse patient populations. However, social and financial factors across this diverse set of 7 hospitals may not be fully representative of hospitals and patients in other regions. If hospitals have different admission thresholds, unobserved severity may differ systematically across hospitals [28]. Second, all 7 hospitals in this study operated using the same EMR platform; it is not known whether access to early admission data, data availability, and EMR adoption stage would be similar in other hospitals. However, this is a common commercial EMR in large institutions. Third, while we derived and validated the model retrospectively in distinct split half datasets, future work to prospectively validate the e-model independently and in other health system settings would be ideal. Finally, the CMS model was developed to evaluate hospital performance using data from the Medicare population using claims data, and not optimized for individual level risk stratification. As such, results of the comparison to CMS model should be interpreted with caution, as the CMS model did not perform well in predicting readmission risk in our population.

## Conclusions

High quality, cost-conscious inpatient care requires hospitals to manage and improve their risk-adjusted 30-day mortality and 30-day readmission rates. Much initial attention has focused on developing disease-specific risk models, but quality and efficiency initiatives are needed for all internal medicine conditions. The multicondition electronic model described in this study performed better than previously published comparators and could be implemented in real-time, suggesting that adult internal medicine patients at highest risk of post-discharge events can be identified early in the course of their hospital stay, when this information is most actionable.

## Additional file

Additional file 1: Table S1. Results of univariate analysis of Risk of 30 Day Readmission or Death^a^. **Table S2.** Multivariate Adjusted Logistic Regression Coefficients For Present on Admission 30-Day Readmission Model. **Table S3.** E-Risk Model Classification Performance for All-Comers 30-Day Readmission Risk Based on Data Available in First 24 Hours^a^. **Figure S1.** Cohort assembly diagram. **Figure S2.** Calibration of Derivation and Validation Cohorts By Quintiles of the Electronic Multicondition Model Predicting 30-Day Readmission and Death. **Figure S3.** Calibration Plot for both Derivation and Validation cohorts.
