# Supplementary material for: Electronic medical record-based multicondition models to predict the risk of 30 day readmission or death among adult medicine patients: validation and comparison to existing models
Source: BMC Med Inform Decis Mak. 2015 May 20;15:39. doi: 10.1186/s12911-015-0162-6 (PMC4474456; doi:10.1186/s12911-015-0162-6)
Supplement: Additional file 1: Table S1. — Results of univariate analysis of Risk of 30 Day Readmission or Deatha. Table S2. Multivariate Adjusted Logistic Regression Coefficients For Present on Admission 30-Day Readmission Model. Table S3. E-Risk Model Classification Performance for All-Comers 30-Day Readmission Risk Based on Data Available in First 24 Hoursa. Figure S1. Cohort assembly diagram. Figure S2. Calibration of Derivation and Validation Cohorts By Quintiles of the Electronic Multicondition Model Predicting 30-Day Readmission and Death. Figure S3. Calibration Plot for both Derivation and Validation cohorts. [file 12911_2015_162_MOESM1_ESM.docx]

**Appendix Table 1. Results of univariate analysis** of Risk of 30 Day Readmission or Death^a^

|  | **Number (%) with RF** | | **Number (%) Without RF**  **With 30-Day Readmission or Mortality** |  |  |
| --- | --- | --- | --- | --- | --- |
| **Risk Factor (RF)** | **Total** | **With 30-Day Readmission or Mortality** |  | **Univariate Odds Ratio (95% CI)** | ***P* Value** |
| Age (mean, range) | 63 (49 - 75) |  |  | 1.01 (1.01 - 1.02) | 0.000 |
| Male | 9 207 (46) | 1 496 (16) | 1 620 (15) | 1.08 (1.00 - 1.16) | 0.054 |
| Payor |  |  |  |  |  |
| Medicare | 8 191 (41) | 1 540 (19) | 1 250 (12) | 1.67 (1.54 - 1.81) | 0.000 |
| Medicaid | 1 371 (7) | 326 (24) | 1 250 (12) | 2.25 (1.96 - 2.58) | 0.000 |
| Elective admission | 2 823 (14) | 277 (10) | 2 839 (17) | 0.54 (0.48 - 0.62) | 0.000 |
| # of Prior ED visits^b^ | 0 (0 - 1) |  |  | 1.06 (1.05 - 1.08) | 0.000 |
| # of Prior Hospitalizations^b^ | 0 (0 - 0) |  |  | 1.31 (1.27 - 1.35) | 0.000 |
| Emergency contacts | 1 (1 - 1) |  |  | 1.21 (1.12 - 1.31) | 0.000 |
| Mental health diagnosis^b^ | 792 (4) | 159 (20) | 2 957 (16%) | 1.37 (1.14 - 1.63) | 0.001 |
| Substance abuse diagnosis^b^ | 993 (5) | 213 (21) | 2 903 (15%) | 1.50 (1.28 - 1.75) | 0.000 |
| Charlson Index^b^ | 0 (0 - 1) |  |  | 1.21 (1.19 - 1.24) | 0.000 |
| Pain | 0 (0 - 4) |  |  | 1.01 (1.00 - 1.02) | 0.137 |
| SpO_2_ (%)<= 94 | 9 743 (49) | 1 702 (17) | 1 414 (14) | 1.30 (1.20 - 1.40) | 0.000 |
| BUN <= 20 mg/dL | 10 995 (55) | 1 403 (13) | 1 713 (19) | 0.61 (0.56 - 0.66) | 0.000 |
| Systolic BP <= 100 mm Hg | 7 015 (35) | 1 239 (18) | 1 877 (15) | 1.25 (1.16 - 1.35) | 0.000 |
| Diastolic BP <= 62 mm Hg | 328 (2) | 88 (27) | 3 028 (16) | 1.99 (1.56 - 2.55) | 0.000 |
| Pulse > 99 | 8 962 (45) | 1 597 (18) | 1 519 (14) | 1.33 (1.24 - 1.44) | 0.000 |
| Sodium > 145 mEq/L | 385 (2) | 105 (27) | 3 011 (15) | 2.05 (1.63 - 2.57) | 0.000 |
| BNP > 2400 / NT-proBNP >18 000 pg/mL | 410 (2) | 122 (30) | 2 994 (15) | 2.32 (1.87 - 2.88) | 0.000 |
| Anion Gap > 18 | 306 (2) | 90 (29) | 3 026 (16) | 2.27 (1.77 - 2.91) | 0.000 |
| Albumin <= 2 g/dL | 209 (1) | 68 (33) | 2 349 (14) | 2.96 (2.21 - 3.97) | 0.000 |
| 2 - 3 g/dL | 2 840 (14) | 699 (25) | 2 349 (14) | 2.01 (1.82 - 2.21) | 0.000 |
| CO_2_ > 30 mmol/L | 600 (3) | 143 (24) | 2 973 (15) | 1.71 (1.41 - 2.07) | 0.000 |
| CPK <= 60 U/L | 2 250 (11) | 459 (20) | 2 657 (15) | 1.44 (1.29 - 1.61) | 0.000 |
| HCT > 35 g/dL | 12736 (64) | 1 727 (14) | 1 389 (20) | 0.64 (0.60 - 0.70) | 0.000 |
| Lymphocytes <= 1.3 x10^9^/L | 5 696 (29) | 1 095 (19) | 2 021 (14) | 1.43 (1.32 - 1.55) | 0.000 |
| MCV > 100 fl | 732 (4) | 174 (24) | 2 942 (15) | 1.71 (1.44 - 2.04) | 0.000 |
| Platelets <= 90 x10^9^/L | 604 (3) | 170 (28) | 2 533 (15) | 2.26 (1.88 - 2.71) | 0.000 |
| > 350 x10^9^/L | 2 104 (11) | 413 (20) | 2 533 (15) | 1.41 (1.25 - 1.58) | 0.000 |
| Protime > 35 sec | 141 (1) | 48 (34) | 3 068 (16) | 2.80 (1.97 - 3.97) | 0.000 |
| TSH > 7 μ IU/mL | 283 (1) | 66 (23) | 3 050 (16) | 1.65 (1.25 - 2.17) | 0.000 |
| AST > 40 U/L | 2 797 (14) | 569 (20) | 2 547 (15) | 1.45 (1.31 - 1.61) | 0.000 |
|  |  |  |  |  |  |

Abbreviations. BUN, blood urea nitrogen; BP, blood pressure; BNP, B-natriuretic peptide; CO_2_, carbon dioxide; CPK, creatinine kinase; TSH, thyroid stimulating hormone; AST, aspartate aminotransferase; HCT, hematocrity; MCV, mean cell volume

^a^ Derivation cohort; composite outcome of 30-day readmission or mortality

^b^ Based on utilization history within 1 year prior to admission

**Appendix Table 2**. Multivariate Adjusted Logistic Regression Coefficients For Present on Admission 30-Day Readmission Model

| **Risk Factor** | **Adjusted Odds Ratio (95% C.I.)** | ***P* value** |
| --- | --- | --- |
| SpO2 <= 94 | 1.10 (1.01 - 1.20) | 0.032 |
| BUN <= 20 | 0.87 (0.80 - 0.96) | 0.004 |
| Pulse > 99 | 1.15 (1.06 - 1.26) | 0.001 |
| Anion Gap > 18 | 1.63 (1.23 - 2.17) | 0.001 |
| Albumin <= 2 | 1.80 (1.30 - 2.50) | 0.000 |
| Albumin 2 - 3 | 1.30 (1.16 - 1.46) | 0.000 |
| CPK <= 60 | 1.23 (1.08 - 1.39) | 0.001 |
| HCT > 35 | 0.82 (0.75 - 0.89) | 0.000 |
| MCV > 100 | 1.32 (1.08 - 1.60) | 0.006 |
| Platelets <= 90 | 1.55 (1.26 - 1.91) | 0.000 |
| Platelets > 350 | 1.19 (1.05 - 1.35) | 0.010 |
| PT > 35 | 1.99 (1.35 - 2.92) | 0.000 |
| TSH > 7 | 1.42 (1.04- 1.93) | 0.027 |
| AST > 40 | 1.15 (1.02 - 1.29) | 0.024 |
| Medicare payor | 1.13 (1.02 - 1.25) | 0.021 |
| Medicaid payor | 1.84 (1.58 - 2.13) | 0.000 |
| Elective admission status | 0.72 (0.62 - 0.82) | 0.000 |
| Prior ED visits in past year | 1.03 (1.02 - 1.05) | 0.000 |
| Prior hospitalizations in past year | 1.19 (1.15 - 1.24) | 0.000 |
| Age | 1.01 (1.01 - 1.02) | 0.003 |
| Charlson Comorbidity index | 1.08 (1.05 - 1.11) | 0.000 |

**Appendix Table 3**. E-Risk Model Classification Performance for All-Comers 30-Day Readmission Risk Based on Data Available in First 24 Hours^a^

| E-Risk Model Classified | Actual Readmission Events | | Total |
| --- | --- | --- | --- |
|  | Readmitted Within 30 Days | Not Readmitted Within 30 Days |  |
| Predicted to Be Readmitted | 1,218^b^ | 4,714 | 5,932 |
| Predicted to Not Be Readmitted | 1,286 | 12,555 | 13,841 |
| Total | 2,504 | 17,269 | 19,773 |

^a^ Based on validation cohort of 19 773 patients and admissions. Classification on basis of predicted probability of event > 0.14, which corresponds to the 70th percentile of the probability distribution among the validation group.

^b^ Corresponds to sensitivity of 49% and positive predictive value of 21%

**Appendix Figure 1.** Cohort assembly diagram

^a^ Parkland Health & Hospital System ( 1 hospital, 12 969 admissions), Texas Health Resources (5 hospitals, 41 026 admissions), and University of Texas Southwestern (1 hospital, 8 441 admissions)

**Appendix Figure 2**. Calibration of Derivation and Validation Cohorts By Quintiles of the Electronic Multicondition Model Predicting 30-Day Readmission and Death.

**Appendix Figure 3**. Calibration Plot for both Derivation and Validation cohorts.
